# Selective Estrogen Receptor Modulator-Like Activities of Herba epimedii Extract and its Interactions With Tamoxifen and Raloxifene in Bone Cells and Tissues

**DOI:** 10.3389/fphar.2020.571598

**Published:** 2021-01-15

**Authors:** Liping Zhou, Ka-Ying Wong, Wenxuan Yu, Christina Chui-Wa Poon, Huihui Xiao, Chi-On Chan, Daniel Kam-Wah Mok, Yan Zhang, Man-Sau Wong

**Affiliations:** ^1^ Cell Therapy Center, Xuanwu Hospital, Capital Medical University, Beijing, China; ^2^ Department of Applied Biology and Chemical Technology, The Hong Kong Polytechnic University, Hong Kong, China; ^3^ State Key Laboratory of Chinese Medicine and Molecular Pharmacology (Incubation), The Hong Kong Polytechnic University Shenzhen Research Institute, Shenzhen, China; ^4^ Longhua Hospital, Shanghai University of Traditional Chinese Medicine, Shanghai, China

**Keywords:** *Herba epimedii*, SERMs, postmenopausal osteoporosis, herb-drug interactions, estrogen receptor

## Abstract

*Herba* *epimedii* (HEP), a kidney-tonifying herb, has been commonly used alone or in formula for strengthening kidney function and treating bone disorders. Its bone protective activity has been demonstrated to be via estrogen receptor (ERs). HEP activates the phosphorylation of ERα in an estrogen response element- (ERE-) dependent manner. We examined the bone protective effects of HEP and its potential interactions with Selective Estrogen Receptor Modulators (SERMs, such as tamoxifen and raloxifene) as they act via the same ERs. Six-month-old mature Sprague Dawley sham-operated (Sham) or ovariectomized (OVX) rats were treated with either vehicle, 17ß-estradiol (1.0 mg/kg.day), tamoxifen (Tamo, 1.0 mg/kg.day), raloxifene (Ralo, 3.0 mg/kg.day), HEP (0.16 g/kg.day), or its combinations with respective SERMs (HEP + Tamo; HEP + Ralo) for 12 weeks. HEP and SERMs as well as their combinations significantly restored changes in bone mineral density (BMD), trabecular bone properties, and bone turnover biomarkers induced by ovarian sex hormone deficiency in ovariectomized rats. Besides the increase in serum estradiol, inhibition on follicle stimulating hormone (FSH) might also be involved in the osteoprotective activities of HEP and SERMs. HEP interacted with SERMs to protect bones from ovarian sex hormone deficiency without altering SERMs’ bone protective activities. HEP neither induced changes in uterus weight nor altered the uterotrophic activity of SERMs in OVX rats. In human osteosarcoma MG-63 cells, HEP-treated serum (HEP-Ts) significantly promoted alkaline phosphatase (ALP) activity like the crude HEP extract did but did not stimulate ERE activity. Our study also reported that biologically activated HEP interacted with SERMs to promote ALP activity without altering the action of SERMs at most of the concentrations tested in MG-63 cells. HEP exerted bone protective activity and the use of HEP did not alter the bone protective activities of SERMs when they were used simultaneously in an estrogen-deficient rat model.

## Introduction

At menopause, circulating estrogen level dramatically declines and results in various distressing symptoms in postmenopausal women. Among these symptoms, osteoporosis is a long-term silent but serious disorder characterized by bone loss and increased risk of fracture, a condition that affects one in three postmenopausal women over the age of 50 and brings a high socioeconomic burden to both their families and the society (Sözen et al., 2017; Shuai et al., 2019). Hormone replacement therapy (HRT) was initially the mainstay of prevention and treatment of postmenopausal bone loss, and moderate exposure to estrogen is associated with significantly increased bone mineral density (BMD) and reduced risk of fracture in postmenopausal women (Going et al., 2003). Unfortunately, as long-term use of estrogen has been shown to be associated with a dramatically increased risk of breast cancer and endometrial cancer (Cagnacci and Venier, 2019), alternative approaches for management of postmenopausal osteoporosis are needed. Indeed, the past decades have witnessed the design and development of Selective Estrogen Receptor Modulators (SERMs) that exert tissue-selective estrogen and antiestrogen effects (Martinkovich et al., 2014). Tamoxifen, the first-generation SERM that is clinically prescribed for treatment of breast cancer, has also been demonstrated to exert osteoprotective activity in both human and animals. However, it increases the risk of endometrium cancer (Tzeng et al., 2015). Raloxifene, the second generation of SERMs approved for treatment of osteoporosis, is an estrogen agonist on bones and helps maintain BMD in postmenopausal women (Cranney et al., 2002). Moreover, raloxifene does not stimulate the growth of uterus tissues and is a potent estrogen antagonist in breast tissues (Martinkovich et al., 2014).

According to the theory of traditional Chinese medicine (TCM), bones are physiologically governed by the “kidney” and problems of the bone are attributed to kidney essence deficiency (Wang and Zhu, 2011). *Herba epimedii* (HEP), a tonifying-kidney herb used by TCM doctors, has been widely used alone for strengthening kidney function and in combination with other herbs for treatment of bone disorders for more than 2000 years (Qin et al., 2005). Many studies have demonstrated the potential antiosteoporotic activity of HEP. Eighty-five clinical trials reported between 2005 and 2016 demonstrated the effectiveness of HEP used alone or in combination with other herbal medicines for managing primary and secondary osteoporosis (Wang et al., 2016). Preclinical studies from our team and others also showed that HEP as a single medicine or in formula reduced bone loss in ovarian sex hormone deficiency animal models without inducing any uterotrophic effects (Chen et al., 2011; Chen et al., 2019). Modern pharmacological study demonstrated that the efficacy of HEP was mediated by the flavonoid phytoestrogens contained in HEP, and these flavonoids promoted osteoblastic functions in rat osteoblastic UMR-106 cells and bone marrow stem cells (Xiao et al., 2014). Previous work by us further demonstrated that the osteogenic actions of HEP flavonoids in UMR-106 cells were estrogen receptor- (ER-) dependent and they could activate ERα in an estrogen response element- (ERE-) dependent manner (Xiao et al., 2014). These results suggest that HEP extract contains phytoestrogens and exerts bone protective activities via ERs.

As the utilization of phytoestrogens and phytoestrogen-rich herbal medicine is increasingly popular among postmenopausal women worldwide (Posadzki et al., 2013), concerns for whether they carry similar benefit-risk profile as estrogen are raised among both the medical community and the postmenopausal women (Patisaul, et al., 2010). Indeed, the safety of using herbal medicine is of particular concern especially for those postmenopausal women who are simultaneously taking the prescribed SERMs. Since the effect of both SERMs and phytoestrogen-rich herbal medicine are mediated by ERs, it is of clinical significance to determine if cotreatment of SERMs with herbal medicine, such as HEP, will increase or decrease their estrogenic effects at target tissue (such as bones) as well as nontarget tissues (such as the uterus).

Therefore, in the present study, we hypothesized that HEP would interact with SERMs in bones. Both mature ovariectomized rats and human osteosarcoma MG-63 cells were employed to systemically study the estrogenic activities of HEP in bones and the uterus as well as the potential interactions between HEP and SERMs (tamoxifen and raloxifene).

## Materials and Methods

### Experimental Design and Animal Treatment

Six-month-old female Sprague Dawley rats (n = 80) were purchased from the Chinese University of Hong Kong. Upon acclimation for 2 weeks, animals were either ovariectomized (OVX) or sham-operated under anesthesia using intraperitoneal injection of ketamine (50 mg/kg) and xylazine (10 mg/kg). Upon a two-week recovery, OVX rats were randomly assigned to one of the following treatments via oral administration: double distilled water as vehicle (OVX), water suspension of 17ß-estradiol (E8875, Sigma; E2, 1.0 mg/kg.day), water suspension of tamoxifen (T5648, Sigma; Tamo, 1.0 mg/kg.day), water suspension of raloxifene (R1402, Sigma; Ralo, 3.0 mg/kg.day), water suspension of HEP (HEP, 0.16 g/kg.day), and its combination with tamoxifen (HEP + Tamo) or raloxifene (HEP + Ralo) for 3 months. Sham-operated rats (n = 10) treated with vehicle were used as control (Sham). Dosages of 17ß-estradiol, tamoxifen, raloxifene, and HEP were in reference to the clinical dosages as stated in our previous studies (Chen et al., 2011; Xie et al., 2005). Procedure of the animal experiment was approved by the Hong Kong Polytechnic University Animal Subjects Ethics Subcommittee (ASESC case: 15-16/31-ABCT-HMRF). During the whole treatment, the animals were pair-fed with a phytoestrogen-free diet (AIN-93M, Research Diets Inc., United States) to remove the influence of phytoestrogens in diet. Their body weight was measured every two weeks. Their urine for 24 h was collected by using metabolic cages one day before euthanasia. At euthanasia, blood was collected from the abdominal aorta and serum samples were prepared after centrifugation at 4,500 rpm for 10 min at 4°C. The uterus was freshly isolated and weighed. The whole left leg and lumbar spine were collected for micro-computed tomography (μCT) analysis.

### Preparation and Quality Control of HEP Extract

The aerial parts of HEP (*Epimedium brevicornu* Maxim) were purchased from Wangcang of Sichuan Province, China, and authenticated by Dr. Chen Sibao at the State Key Laboratory of Chinese Medicine and Molecular Pharmacology (Incubation) in Shenzhen (No.: SZ2016HEP01). Icariin, the most abundant active compound in HEP, was selected for evaluation of quality by high performance liquid chromatography (HPLC) assay to ensure the fulfillment of requirement of the China Pharmacopoeia and/or the Hong Kong Chinese Materia Medica Standard. Upon authentication, extracts of HEP were prepared by Xi’an Pincredit Bio-tech Co., Ltd., using a traditional method. Briefly, HEP was soaked in 10 volumes of water (v/w) for 20 min and then boiled in 1 h for three times (Yang et al., 2013). The extracts were dried by lyophilization and stored at −80°C. The amount of icariin in the HEP extracts was determined by liquid chromatography-mass spectrometry (LC-MS).

### Biochemical Analysis of Serum and Urine

Calcium (Ca) and phosphorus (P) levels in serum and urine as well as urinary level of creatinine were measured by Arsenazo III UV method with an automatic analyzer HITACHI7100. The kits were purchased from Shanghai Kehua Bio-Engineering Co., Ltd. (Shanghai, China). Urinary deoxypyridinoline (DPD) was determined by an enzyme immunoassay DPD EIA kit (QUIDEL Corporation, United States) and normalized by urinary creatinine. The diestrus stage of the estrous cycle in the sham group was used in order to reduce the fluctuation of sexual hormone in the proestrus and estrus stages.

### Micro-Computed Tomography Analysis

Bone properties of the trabecular bone at the proximal tibia and distal femur as well as lumbar vertebra were determined by micro-CT (μCT40, Scanco Medical, Switzerland) as previously described (Zhou et al., 2018). The source energy selected for this study was 70 KVp and 114 μA with a resolution of 21 μm. Approximately 200 slices were done for each scan. The distal/proximal were defined as 4.2 and 2.2 mm away from the femur/tibia head. Scanning was done at the metaphyseal area that was located 0.63 mm below the lowest point of the epiphyseal growth plate and extended 2.0 mm in the proximal direction. Bone mineral density (BMD, mg HA/ccm) and bone morphometric properties, including bone volume over total volume (BV/TV), connectivity density (Conn.D, 1/mm^3^), structure model index (SMI), trabecular bone number (Tb.N, mm^−1^), trabecular bone thickness (Tb.Th, mm), and trabecular bone separation (Tb.Sp, mm), were evaluated by contoured volume-of-interest (VOI) images.

### Preparation of HEP-Treated Serum

HEP is a mixture of phytoestrogens that might not be completely bioavailable and activities of them might require biological activation *in vivo*. HEP-treated serum (HEP-Ts) was prepared and applied to evaluate the direct estrogenic actions of HEP *in vitro*. OVX rats were given vehicle or HEP at 1.6 g/kg.day for 3 consecutive days and pair-fed with the phytoestrogen-free AIN-93 diet. Upon the last oral administration on Day 3, the rats were fasted overnight and orally fed with HEP one more time in the following morning. Rats were euthanized an hour after administration. Blood was collected from the abdominal aorta and serum was prepared and stored at -80 °C. Liquid chromatography-mass spectrometry (LC-MS) analysis of the serum was performed to confirm the presence of major chemical markers from HEP. Methanol extract of serum was prepared and extract of 1 ml serum was dissolved in 1 ml of ethanol and the concentration of this solution was defined as “1”. Microsep^TM^ Advance Centrifugal Device (3K) was used to remove small molecules including steroid in the serum extract and the solution was sterilized with 0.22 μm membrane. Various dilutions of the serum extract (10^−5^, 10^−4^, 10^−3^, and 10^−2^) were used for *in vitro* study.

### Cell Culture and ALP Assay

Human osteosarcoma MG-63 cells (ATCC® CRL-1427^TM^, passage 3–10) were in monolayer in Minimum Essential Medium (MEM, Gibco) containing 100 U/ml penicillin, 100ug/ml streptomycin (Invitrogen, Carlsbad), and 10% Fetal Bovine Serum (FBS, Gibco). The cultures were maintained in an incubator at 37°C in a humidified atmosphere of 95% O_2_ and 5% CO_2_. Cells were cultured in 24-well plate (20,000 cell/well) with phenol red-free (PRF) medium containing 5% charcoal stripped fetal bovine serum (cs-FBS) before treatment with vehicle, estradiol (10^−8^ M), crude HEP extract (10–400 μg/ml), HEP-Ts (10^−4^–10^-2^ dilution), tamoxifen (10^−12^ to 10^−6^ M), raloxifene (10^−12^ to 10^−6^ M), or their combinations with HEP-Ts at the optimal concentration in PRF medium for 48 h. Upon treatment, 100 μl of Passive Lysis Buffer was added to each well to lyse cells. The ALP activity of cell lysate was measured by a LabAssayTM ALP kit (Wako, Japan) following the manufacturer’s instruction. Total protein concentrations of the cell lysate were measured via Bradford method to normalize ALP activity.

### Statistical Analysis

Data were reported as mean ± SEM. Intergroup differences of *in vivo* experiment were analyzed by one-way ANOVA with Tukey’s as post hoc test. Intergroup differences of *in vitro* experiment were analyzed by t-test. Interactions between herb and drug were analyzed by two-way ANOVA with Bonferroni as post hoc test. A value of *p* < 0.05 was considered statistically significant.

## Results

### Preparation and Quantification of HEP Extract

**FIGURE 1:**
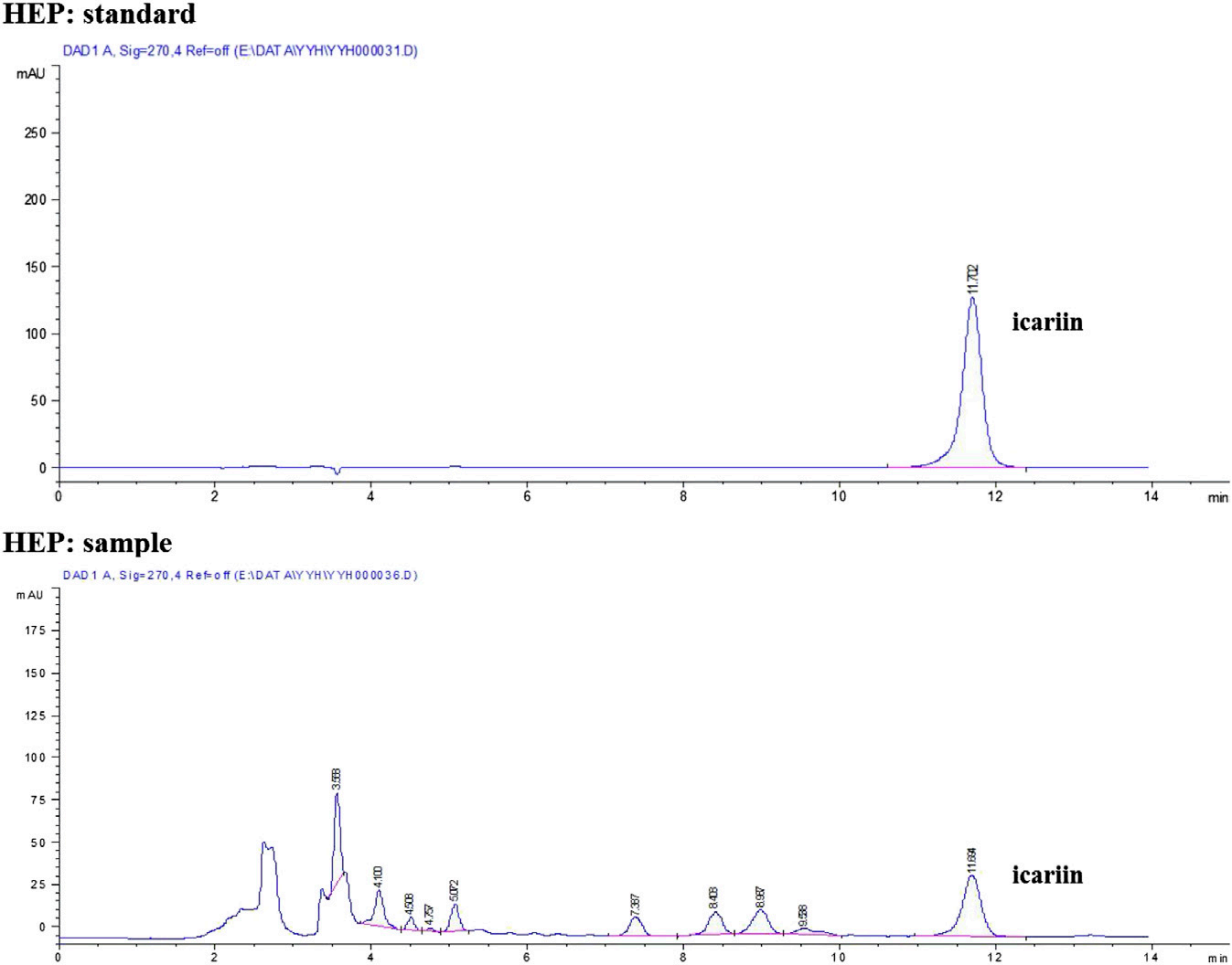
HPLC fingerprint of HEP raw herb.

HPLC profile confirmed that the quality of all raw herbs had fulfilled the respective requirements (Figure 1). According to results of LC-MS, the amount of icariin was 8.377 mg in 1 g of dried HEP extract.

#### Effects of HEP Alone and its Combinations With Tamoxifen or Raloxifene on Body Weight, Uterus Weight and Biochemical Parameters in OVX Rats

The effects of HEP alone and its combinations with SERMs on body weight gain and uterus weight as well as serum and urine biochemical parameters were measured in mature OVX rats after oral administration for 3 months. The body weight of OVX rats was significantly elevated in response to OVX (vs. Sham) and the increase was dramatically reversed by treatment with E2, tamoxifen, raloxifene, and their combinations with HEP, but not HEP alone (Table 1, *p* < 0.001 vs. OVX). According to the results of two-way ANOVA, there were no interactions between HEP and either tamoxifen or raloxifene in suppressing body weight gain in OVX rats. Uterus index expressed as the ratio of uterus to body weight was compared between groups for the assessment of uterotrophic effect of different treatments in OVX rats. A dramatic decline in uterus index was observed in rats upon OVX (Table 1, *p* < 0.001 vs. sham), indicating successful OVX operation. Estradiol, tamoxifen, and raloxifene significantly increased the uterus index in OVX rats (Table 1, *p* < 0.01 vs. OVX). In contrast, treatment with HEP alone did not induce any changes in uterus index in OVX rats. Combinations of HEP with tamoxifen (HEP + Tamo) and raloxifene (HEP + Ralo) significantly increased uterus weight in OVX rats, respectively (Table 1, *p* < 0.05 vs OVX). Two-way ANOVA indicated interaction between HEP and tamoxifen (HEP x Tamo: *p* = 0.0457), but not between HEP and raloxifene. No significant changes in serum Ca, P or urine Ca, P excretion were observed in OVX rats treated with E2, tamoxifen, raloxifene, HEP alone, or the combinations of HEP and SERMs in OVX rats (Table 1). Two-way ANOVA indicated HEP interacted with raloxifene in regulating urine excretion of Ca and P in OVX rats (HEP x Ralo: *p* = 0.0322 for urinary Ca, *p* = 0.0014 for urinary P).

**TABLE 1:** Effects of estradiol, tamoxifen, raloxifene, HEP, and their combinations on body weight, uterus weight, and biochemical parameters in OVX rats.

Group	Body weight (% to baseline)	Uterus index (mg/g)	Serum biochemistry			
			Serum Ca (mg/L)	Serum P (mg/L)	Urinary Ca/Cr (mg/mg)	Urinary P/Cr (mg/mg)
Sham	11.13 ± 1.62	1.77 ± 0.12	99.92 ± 2.39	46.99 ± 1.31	0.091 ± 0.015	0.30 ± 0.06
OVX	18.76 ± 0.64^*^	0.33 ± 0.02^***^	96.31 ± 1.55	46.19 ± 1.31	0.078 ± 0.008	0.26 ± 0.03
E2	0.17 ± 1.84^^^^^	1.58 ± 0.04^^^^^	99.44 ± 2.92	48.05 ± 3.33	0.100 ± 0.011	0.30 ± 0.03
Tamo	−0.26 ± 1.76^^^^^	0.70 ± 0.02^^^^	91.23 ± 2.26	47.39 ± 1.77	0.082 ± 0.011	0.34 ± 0.03
Ralo	5.43 ± 2.16^^^^^	0.63 ± 0.03^^^^	94.35 ± 1.95	45.96 ± 1.31	0.099 ± 0.011	0.41 ± 0.03
HEP	18.99 ± 1.65	0.45 ± 0.03	94.79 ± 1.13	46.71 ± 1.28	0.103 ± 0.007	0.37 ± 0.02
HEP + Tamo	−3.67 ± 1.60^^^^; ###^	0.64 ± 0.05^^^^	93.63 ± 1.46	50.08 ± 1.67	0.085 ± 0.013	0.34 ± 0.03
HEP + Ralo	5.09 ± 0.86^^^^; ###^	0.60 ± 0.04^^^	95.06 ± 0.69	48.63 ± 2.02	0.085 ± 0.009	0.32 ± 0.03
*p* value of two-way ANOVA						
Tamo	<0.0001	<0.0001	0.0570	0.0778	0.4842	0.3398
HEP	0.4017	0.2773	0.7834	0.4747	0.1723	0.0573
HEP ✕ Tamo	0.3119	0.0457	0.2235	0.3083	0.2716	0.0570
Ralo	<0.0001	<0.0001	0.5564	0.3844	0.8655	0.0872
HEP	0.6950	0.1493	0.7782	0.4759	0.5328	0.8161
HEP ✕ Ralo	0.5741	0.0618	0.4392	0.3085	0.0322	0.0014

After two weeks of recovery, 6-month-old mature Sprague Dawley sham-operated (Sham) or ovariectomized (OVX) rats were treated with either vehicle, 17ß-estradiol (1.0 mg/kg.day), tamoxifen (Tamo, 1.0 mg/kg.day), raloxifene (Ralo, 3.0 mg/kg.day), HEP (0.16 g/kg.day), or its combinations with respective SERMs for 12 weeks. Body weight was measured every two weeks. At sacrifice, uterus was freshly isolated and weighed. Ratio of uterus weight (mg) to body weight was defined as uterus index. Calcium (Ca) and phosphorus (P) level in serum and urine as well as urinary level of creatinine were measured by Arsenazo III UV method with an automatic analyzer HITACHI7100. Data were expressed as mean ± SEM. n = 7-9 rats per group. Differences between groups were determined by one-way ANOVA followed by Tukey’s test for post hoc comparison. Interactions between HEP and SERMs were determined by two-way ANOVA followed by Bonferroni test as post test. ^*^p < 0.05, ^***^p < 0.001 vs. sham; ^^^p < 0.05, ^^^^p < 0.01, ^^^^^p < 0.001 vs. OVX; ^###^p < 0.001 vs. HEP. Ca: calcium; Cr: creatinine.

#### Effects of HEP Alone and in Combination With SERMs on Serum Level of Reproductive Hormones in OVX Rats

**FIGURE 2:**
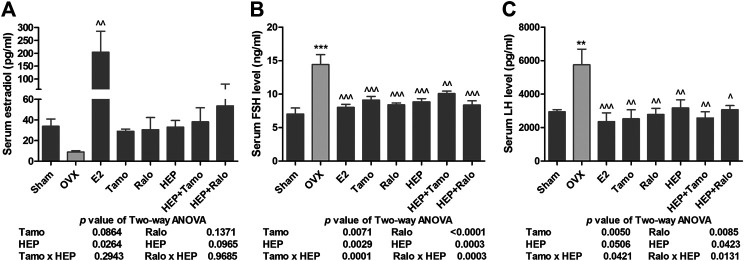
Effects of HEP, SERMs alone, and their combinations on serum level of reproductive hormones in mature ovariectomized rats. Six-month-old mature Sprague Dawley sham-operated (Sham) or ovariectomized (OVX) rats were treated with either vehicle, 17ß-estradiol (1.0 mg/kg.day), tamoxifen (Tamo, 1.0 mg/kg.day), raloxifene (Ralo, 3.0 mg/kg.day), HEP (0.16 g/kg.day), or the combinations of HEP with respective SERMs for 12 weeks. At euthanasia, serum was collected and serum levels of estradiol, follicle stimulating hormone (FSH), and luteinizing hormone (LH) were measured by EIA or ELISA kits. **(A)** Circulating estradiol level. **(B)** Circulating FSH level. **(C)** Circulating LH level. Data were expressed as mean ± SEM. n = 6 to 12. Differences between groups were determined by one-way ANOVA followed by Tukey’s test for post hoc comparison. Interactions between HEP and SERMs were determined by two-way ANOVA followed by Bonferroni test as post test. ^***^ *p* < 0.001 vs. sham; ^^^ *p* < 0.05, ^^^^ *p* < 0.01 vs. OVX, ^^^^^ *p* < 0.001 vs. OVX.

Recent studies have suggested that the increased FSH level, in addition to the reduced production of estradiol, might account for bone loss during menopause (Sun et al., 2006; García-Martín et al., 2012). The present study indicated that serum estradiol was markedly decreased in OVX rats and this decrease was accompanied by a significant increase in circulating levels of FSH and LH (Figure 2, *p <* 0.01 vs. sham). As expected, treatment of OVX rats with estradiol significantly reversed the serum level of estradiol and completely suppressed the FSH and LH levels to a level comparable to those of sham rats (Figure 2, *p <* 0.01 vs. OVX). Increases in serum estradiol were also observed in OVX rats treated with tamoxifen, raloxifene, and HEP as well as their combinations but the changes did not reach statistical significance (Figure 2A, vs. OVX). Similar to estrogen, tamoxifen, raloxifene, HEP alone, and their combinations all dramatically reduced the increase in both circulating FSH and LH levels in OVX rats (Figures 2B,C, *p* < 0.05 vs. OVX). No statistical differences in reproductive hormones level were detected between OVX rats treated with SERMs and OVX rats treated with respective SERMs in combination with HEP, indicating HEP did not alter the actions of SERMs on reproductive hormones. Two-way ANOVA analysis indicated HEP and SERMs interacted to suppress FSH and LH levels (HEP ✕ tamoxifen, FSH: *p* = 0.0001, LH: *p* = 0.0421; HEP ✕ raloxifene, FSH: *p* = 0.0003, LH: *p* = 0.0131), but their interaction did not restore the estradiol level in OVX rats.

#### Effects of HEP Alone and in Combination With SERMs on Bone Turnover Biomarkers in OVX Rats

**FIGURE 3:**
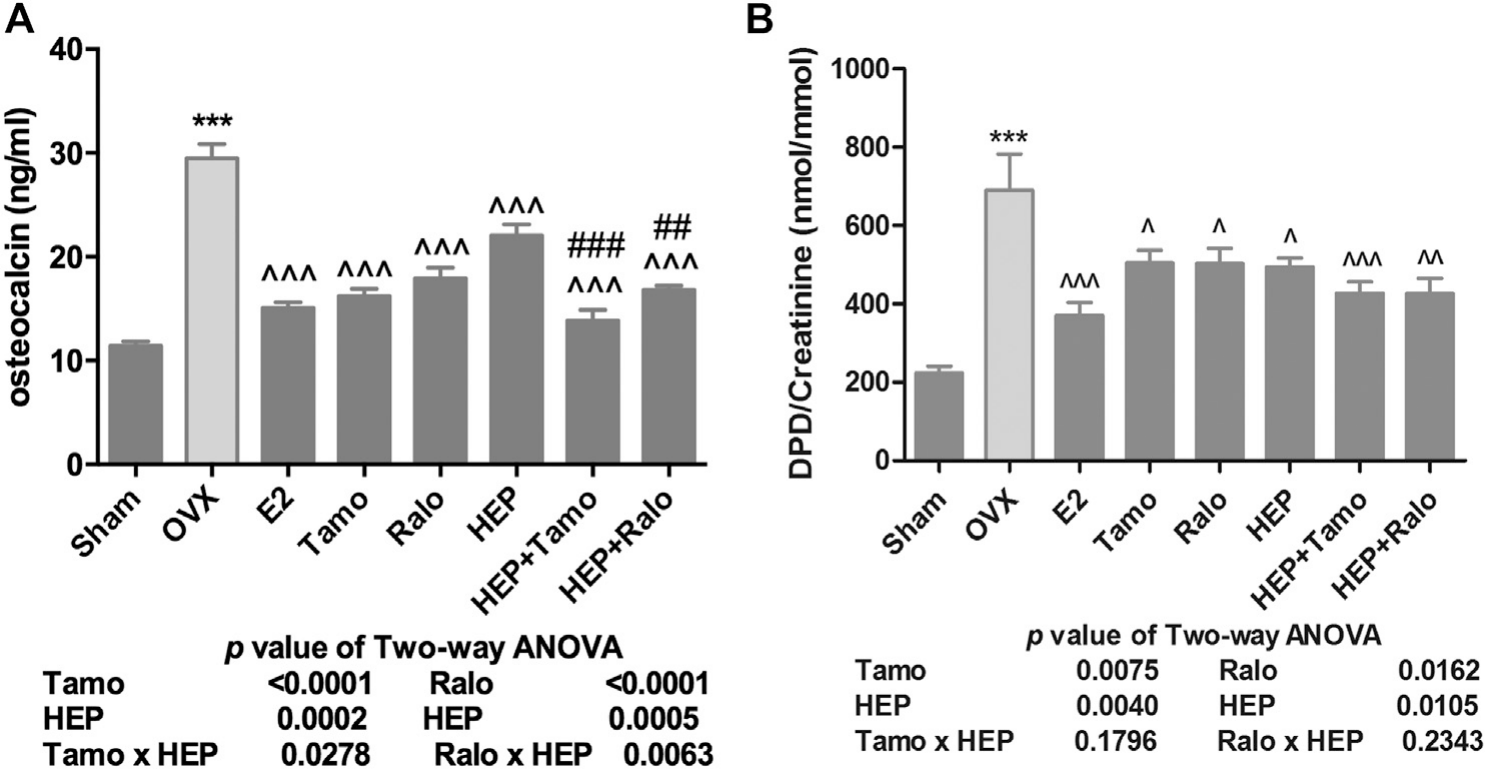
Effects of HEP, SERMs alone, and their combinations on bone mineral density (BMD) and bone structure in mature ovariectomized rats. Six-month-old mature Sprague Dawley sham-operated (Sham) or ovariectomized (OVX) rats were treated with either vehicle, 17ß-estradiol (1.0 mg/kg.day), tamoxifen (Tamo, 1.0 mg/kg.day), raloxifene (Ralo, 3.0 mg/kg.day), HEP (0.16 g/kg.day), or the combination of HEP with respective SERMs for 12 weeks. At euthanasia, the whole left leg and lumbar spine were collected. Bone mineral density at the distal femur, proximal tibia, and lumbar vertebra **(A)** and bone microstructure at proximal tibia **(B)** were measured by micro-CT. Data were expressed as mean ± SEM. n = 5 to 12. Differences between groups were determined by one-way ANOVA followed by Tukey’s test for post hoc comparison. Interactions between HEP and SERMs were determined by two-way ANOVA followed by Bonferroni test as post test.^***^ *p* < 0.001 vs. sham; ^^^ *p* < 0.05, ^^^^^ *p* < 0.001 vs. OVX; ^##^ *p* < 0.01, ^###^ *p* < 0.001 vs. HEP.

Serum osteocalcin (OCN) and urinary deoxypyridinoline (DPD), which were bone formation and bone resorption markers, respectively, were measured (Tang et al., 2016; Zoch et al., 2016). As shown in Figure 3, both serum OCN and urinary DPD increased significantly in OVX rats (*p* < 0.001 vs. sham) while treatment of OVX rats with 17ß-estradiol significantly restored changes in OCN and DPD (*p* < 0.001 vs. OVX). Treatment with both tamoxifen and raloxifene also significantly suppressed the increase in OCN and DPD levels in OVX rats (*p* < 0.001 vs. OVX). However, treatment with HEP alone only significantly restored ovarian sex hormone deficiency-induced changes in OCN level (Figure 3A, *p* < 0.05 vs. OVX), but not urinary DPD (Figure 3B, vs. OVX), in OVX rats. Cotreatment of OVX rats with HEP and SERMs also markedly reduced both serum OCN and urinary DPD (*p* < 0.01 vs. OVX). Two-way ANOVA analysis suggested there were no interactions between HEP and tamoxifen while HEP interacted with raloxifene to suppress serum OCN (HEP ✕ raloxifene, *p* = 0.0334).

#### Effects of HEP Alone and in Combination With SERMs on Bone Mineral Density (BMD) and Trabecular Bone Properties in OVX Rats

**FIGURE 4:**
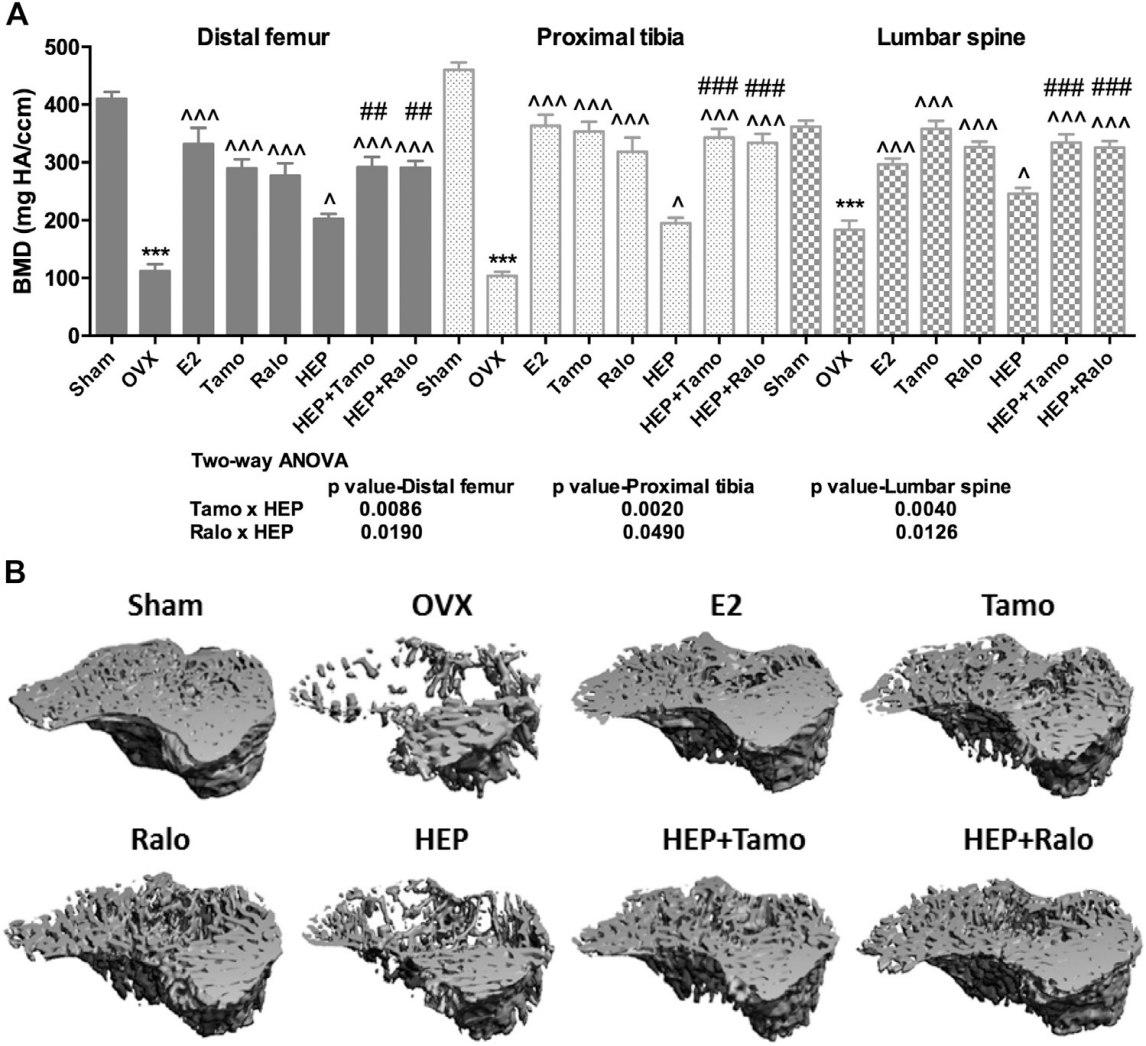
Effects of HEP, SERMs alone, and their combinations on bone turnover biomarkers in mature ovariectomized rats. Upon treatment, serum levels of osteocalcin (OCN) and urinary deoxypyridinoline (DPD) were measured by ELISA kits following the manufacturers’ instruction. **(A)** Serum level of OCN. **(B)** Urinary level of DPD. Data were expressed as mean ± SEM. n = 5 to 12. Differences between groups were determined by one-way ANOVA followed by Tukey’s test for post hoc comparison. Interactions between HEP and SERMs were determined by two-way ANOVA followed by Bonferroni test as post test. ^***^ *p* < 0.001 vs. sham; ^^^ *p* < 0.05, ^^^^ *p* < 0.01, ^^^^^ *p* < 0.001 vs. OVX; ^##^ *p* < 0.01, ^###^ *p* < 0.001 vs. HEP.

Micro-computed tomography (micro-CT) was performed to evaluate the effects of HEP alone and its combinations with SERMs on bone properties in OVX rats. Figure 4A clearly showed that OVX significantly decreased BMD at the distal femur, proximal tibia, and lumbar vertebra in rats (*p* < 0.001 vs. sham). All treatments significantly increased BMD at the three sites measured in OVX rats (*p* < 0.05 vs. OVX). Estrogen, HEP, SERMs, and their combinations dramatically protected bone from ovarian sex hormone deficiency-induced deteriorations in microstructure of the proximal tibia (Figure 4B) and trabecular bone properties at all the three sites (Table 2). Unsurprisingly, bone volume/total volume (BV/TV), connectivity density (Conn.D), trabecular bone number (Tb.N), and trabecular thickness (Tb.Th) significantly decreased in response to OVX while structure model index (SMI) and trabecular separation (Tb.Sp) significantly increased at all three bone sites of OVX rats (*p* < 0.05 vs. sham). Treatment of OVX rats with estradiol, SERMs, HEP alone, and their combinations significantly increased BV/TV, Conn.D, Tb.N, and Tb.Th and decreased SMI and Tb.Sp of bone at all three sites (*p* < 0.05 vs. OVX). No significant differences were found in BMD and bone properties at all three sites between OVX rats treated with SERMs alone and those treated with the combination of HEP and respective SERMs, indicating cotreatment with HEP did not significantly alter the bone protective activities of SERMs. Two-way ANOVA analysis indicated that HEP interacted with tamoxifen and with raloxifene on increasing BMD at all the three sites in OVX rats (Figure 4A, HEP ✕ tamoxifen, distal femur: *p* = 0.0086, proximal tibia: *p* = 0.0020, lumbar vertebra: *p* = 0.0040; HEP ✕ raloxifene, distal femur: *p* = 0.0190, proximal tibia: *p* = 0.0490, lumbar vertebra: *p* = 0.0126). Moreover, HEP interacted with both SERMs to improve trabecular bone properties at all the three sites in OVX rats (Table 2, *p* < 0.05).

**TABLE 2:** Effects of estradiol, tamoxifen, raloxifene, HEP, and their combinations on trabecular bone properties of OVX rats.

	Trabecular bone properties (distal femur)					
	BV/TV	Conn.D 1/mm^3^	SMI	Tb.N mm^−1^	Tb.Th mm	Tb.Sp mm
Sham	0.49 ± 0.02	44.04 ± 0.92	-0.97 ± 0.22	4.03 ± 0.10	0.16 ± 0.005	0.21 ± 0.01
OVX	0.07 ± 0.01^***^	8.19 ± 1.45^***^	2.21 ± 0.13^***^	1.46 ± 0.04^***^	0.10 ± 0.002^***^	0.67 ± 0.017^***^
E2	0.37 ± 0.04^^^^^	34.23 ± 2.07^^^^^	-0.53 ± 0.44^^^^^	2.80 ± 0.29^^^^^	0.14 ± 0.009^^^^^	0.38 ± 0.049^^^^^
Tamo	0.29 ± 0.02^^^^^	35.67 ± 1.74^^^^^	0.45 ± 0.15^^^^^	2.72 ± 0.21^^^^^	0.13 ± 0.004^^^^^	0.37 ± 0.037^^^^^
Ralo	0.25 ± 0.02^^^^^	27.59 ± 2.37^^^^^	0.35 ± 0.16^^^^^	2.45 ± 0.15^^^^^	0.13 ± 0.005^^^^	0.45 ± 0.029^^^^^
HEP	0.24 ± 0.02^^^	20.27 ± 1.57^^^^^	1.01 ± 0.14^^^	2.18 ± 0.10	0.13 ± 0.002	0.46 ± 0.027^^^^
HEP + Tamo	0.32 ± 0.02^^^^;#^	33.85 ± 1.71^^^^;###^	0.79 ±.0.16^^^^^	2.74 ± 0.17^^^^^	0.13 ± 0.004^^^^^	0.32 ± 0.019^^^^^
HEP + Ralo	0.36 ± 0.04^^^^^	28.84 ± 1.40^^^^;#^	0.58 ± 0.18^^^^^	2.42 ± 0.10^^^^^	0.14 ± 0.004^^^^	0.40 ± 0.012^^^^^
*p* value of two-way ANOVA						
Tamo	<0.0001	<0.0001	<0.0001	<0.0001	0.0023	<0.0001
HEP	<0.0001	0.0055	0.0106	0.0388	0.0010	0.0001
HEP ✕ Tamo	0.0024	0.0004	0.0004	0.0498	0.0029	0.0087
Ralo	<0.0001	<0.0001	<0.0001	<0.0001	0.0003	<0.0001
HEP	<0.0001	0.0012	0.0202	0.0107	0.0014	<0.0001
HEP ✕ Ralo	0.2932	0.0062	0.0002	0.0067	0.0058	0.0035

	**Trabecular bone properties (proximal tibia)**					
	BV/TV	Conn.D 1/mm^3^	SMI	Tb.N mm^−1^	Tb.Th mm	Tb.Sp mm
Sham	0.60 ± 0.02	55.46 ± 2.41	−2.04 ± 0.39	4.67 ± 0.04	0.16 ± 0.005	0.15 ± 0.005
OVX	0.07 ± 0.01^***^	5.70 ± 0.99^***^	2.65 ± 0.05^***^	1.29 ± 0.11^***^	0.11 ± 0.002^***^	0.74 ± 0.071^***^
E2	0.40 ± 0.02^^^^^	44.74 ± 1.87^^^^^	0.12 ± 0.37^^^^^	3.62 ± 0.09^^^^^	0.15 ± 0.006^^^	0.23 ± 0.013^^^^^
Tamo	0.37 ± 0.02^^^^^	47.87 ± 2.08^^^^^	0.49 ± 0.33^^^^^	3.64 ± 0.15^^^^^	0.14 ± 0.005^^^^^	0.22 ± 0.014^^^^^
Ralo	0.37 ± 0.03^^^^^	39.02 ± 2.51^^^^^	0.87 ± 0.17^^^^^	3.40 ± 0.13^^^^^	0.14 ± 0.006^^^^	0.30 ± 0.062^^^^^
HEP	0.18 ± 0.01^^^	16.65 ± 1.77^^^	2.24 ± 0.08	2.16 ± 0.09^^^^	0.13 ± 0.003	0.56 ± 0.050^^^
HEP + Tamo	0.38 ± 0.01^^^^;###^	41.33 ± 3.24^^^^;###^	0.79 ± 0.18^^^^;##^	3.40 ± 0.11^^^^;###^	0.14 ± 0.005^^^^^	0.24 ± 0.013^^^^;###^
HEP + Ralo	0.34 ± 0.02^^^^;###^	36.84 ± 2.14^^^^;###^	0.81 ± 0.19^^^^;##^	3.23 ± 0.13^^^^;###^	0.14 ± 0.004^^^^	0.26 ± 0.015^^^^;###^
*p* value of two-way ANOVA						
Tamo	<0.0001	0.0017	<0.0001	<0.0001	0.0002	<0.0001
HEP	0.0030	0.3781	0.7821	0.0150	0.0057	0.3530
HEP✕Tamo	0.0033	0.0017	0.1106	<0.0001	0.0447	0.0205
Ralo	0.0023	<0.0001	<0.0001	0.002	0.0001	<0.0001
HEP	0.0610	0.0348	0.1110	0.0071	0.0514	0.0699
HEP✕ Ralo	0.0023	0.0029	0.2162	<0.0001	0.0073	0.0217

	**Trabecular bone properties (lumbar spine)**					
	BV/TV	Conn.D 1/mm^3^	SMI	Tb.N mm^−1^	Tb.Th mm	Tb.Sp mm
Sham	0.41 ± 0.017	33.97 ± 2.70	0.13 ± 0.122	3.36 ± 0.059	0.13 ± 0.003	0.24 ± 0.006
OVX	0.14 ± 0.014^***^	10.54 ± 1.99^***^	2.06 ± 0.109^***^	1.96 ± 0.080^***^	0.10 ± 0.001^***^	0.47 ± 0.026^***^
E2	0.34 ± 0.048^^^^^	25.70 ± 2.34^^^^^	0.57 ± 0.339^^^^	2.84 ± 0.160^^^^^	0.12 ± 0.008	0.32 ± 0.021^^^^^
Tamo	0.41 ± 0.022^^^^	33.00 ± 2.31^^^^^	0.11 ± 0.181^^^^^	3.32 ± 0.090^^^^^	0.14 ± 0.005^^^^^	0.27 ± 0.011^^^^^
Ralo	0.35 ± 0.019^^^^	29.30 ± 1.51^^^^^	0.76 ± 0.197^^^^	3.07 ± 0.080^^^^^	0.13 ± 0.002^^^	0.28 ± 0.010^^^^^
HEP	0.25 ± 0.013^^^^	20.46 ± 1.84^^^	1.26 ± 0.111^^^	2.52 ± 0.053^^^	0.12 ± 0.003	0.40 ± 0.016^^^^^
HEP + Tamo	0.37 ± 0.021^^^^;##^	26.84 ± 1.21^^^^^	0.30 ± 0.183^^^^;#^	3.10 ± 0.080^^^^;##^	0.13 ± 0.004^^^^	0.29 ± 0.009^^^^;###^
HEP + Ralo	0.35 ± 0.023^^^^;#^	25.86 ± 1.40^^^^^	0.51 ± 0.155^^^^^	3.01 ± 0.113^^^^;##^	0.13 ± 0.003^^^^	0.29 ± 0.013^^^^;###^
*p* value of two-way ANOVA						
Tamo	<0.0001	<0.0001	<0.0001	<0.0001	<0.0001	<0.0001
HEP	0.0958	0.3465	0.0663	0.0460	0.2017	0.1844
HEP ✕ Tamo	0.0007	0.0005	0.0056	<0.0001	0.0069	0.0162
Ralo	<0.0001	<0.0001	<0.0001	<0.0001	<0.0001	<0.0001
HEP	0.0048	0.0666	0.0044	0.0120	0.0031	0.0980
HEP ✕ Ralo	0.0078	0.0006	0.1100	0.0022	0.0046	0.0279

After two weeks of recovery, 6-month-old mature Sprague Dawley sham-operated (Sham) or ovariectomized (OVX) rats were treated with either vehicle, 17ß-estradiol (1.0 mg/kg.day), tamoxifen (Tamo, 1.0 mg/kg.day), raloxifene (Ralo, 3.0 mg/kg.day), HEP (0.16 g/kg.day), or its combinations with respective SERMs for 12 weeks. Bone properties of trabecular bone at proximal tibia and distal femur as well as lumbar vertebra were determined by micro-CT (μCT40, Scanco Medical, Switzerland). Data were expressed as mean ± SEM. n = 7-9 rats per group. Differences between groups were determined by one-way ANOVA followed by Tukey’s test for post hoc comparison. Interactions between HEP and SERMs were determined by two-way ANOVA followed by Bonferroni test as post test. ^***^p < 0.001 vs. sham; ^^^p < 0.05, ^^^^p < 0.01, ^^^^^p < 0.001 vs. OVX; ^#^p < 0.05, ^##^p < 0.01, ^###^p < 0.001 vs. HEP.

#### Effects of HEP Alone and in Combination With SERMs on Estrogenic Activities in Human Osteosarcoma MG-63 Cells

**FIGURE 5:**
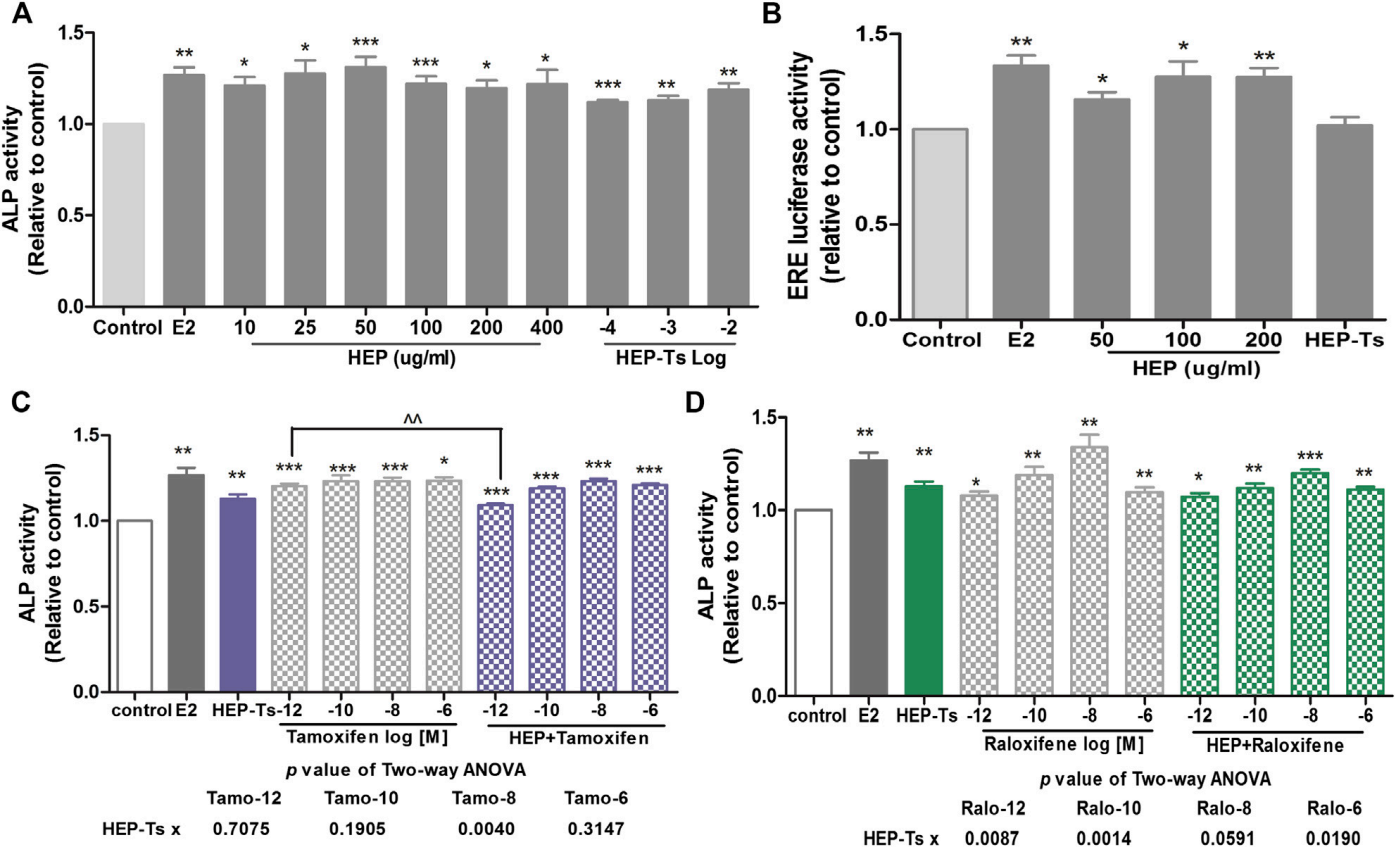
Direct estrogenic effects of HEP, SERMs alone, and their combinations on ALP activity and ERE-luciferase activity in human osteosarcoma MG-63 cells. Human osteosarcoma MG-63 cells were routinely cultured and treated with HEP (crude HEP and HEP-treated serum), tamoxifen, raloxifene, or their combinations for 48 h. Upon treatment, ALP activity and estrogen response element- (ERE-) luciferase activity were measured by ALP assay and Dual Luciferase® Reporter Assay System, respectively. Results were expressed as ratio to control. n = 3 or more. Differences between groups were determined by independent t-test. Interactions between HEP and SERMs were determined by two-way ANOVA followed by Bonferroni test as post test. ^*^ *p* < 0.05, ^**^ *p* < 0.01, ^***^ *p* < 0.001 vs. control; ^^^ *p* < 0.05 vs. SERM alone; ^^^^ *p* < 0.01 vs. tamoxifen alone.

Both crude HEP extract and biologically activated HEP (HEP-Ts) in the form of HEP-treated serum were applied to human osteosarcoma MG-63 cells for evaluation of the direct estrogenic activity of HEP. Table 3 shows the presence of major polyphenol and alkaloid groups in the HEP crude extract as well as in the treated serum, suggesting that HEP extract and its metabolites had been absorbed and transported in rat circulation. Both crude HEP and HEP-Ts notably increased alkaline phosphatase (ALP) activities in MG-63 cells in a dose-dependent manner (Figure 5A, *p* < 0.05 vs. control). Crude HEP at 5 μg/ml to 400 μg/ml exerted potent stimulatory effect on ALP activities in MG-63 cells. HEP-Ts also dose-dependently induced ALP activities and the most potent stimulatory effect appeared at 10^-3^ dilution. Upon treatment with crude HEP not HEP-Ts, ERE-luciferase activities significantly increased in MG-63 cells (Figure 5B, *p* < 0.05). These results suggested that HEP directly exerted estrogen-like activities in MG-63 cells while the estrogenic activities were different between crude HEP and biologically activated HEP. To best mimic animal conditions, biologically activated HEP was used to study the herb-drug interaction. Both tamoxifen and raloxifene from 10^−12^ to 10^−6^ M dramatically increased ALP activities in MG-63 cells (Figures 5C and 5D, *p* < 0.05). Upon their cotreatment for 48 h, HEP-Ts at 10^−3^ dilution significantly weakened the stimulatory effect of tamoxifen on ALP activities in MG-63 cells (Figure 5C, *p* < 0.01, Tamo vs. HEP + Tamo). Two-way ANOVA analysis of their effects on ALP activities indicated that HEP-Ts interacted with raloxifene from 10^−12^ to 10^−6^ M (HEP-Ts x ralo: *p* = 0.0087 at 10^−12^ M, *p* = 0.0014 at 10^−10^ M, *p* = 0.0190 at 10^−6^ M) and with tamoxifen at 10^−8^ M (HEP-Ts x Tamo: *p* = 0.0040 at 10^−8^ M).

**TABLE 3:** Major polyphenol and alkaloids detected in HEP extract and HEP-treated serum by LC-MS in ESI (+) and ESI (-) mode.

Possible ID	Empirical formula	Charge	Expected m/z	HEP extract			HEP-treated serum		
				R.T	Expt m/z	Mass error	R.T	Expt m/z	Mass error
**ESI (+) mode**									
Berberine	C20H17NO4	[M + H]+	336.1230	11.59	336.1219	−3.27	11.6	336.1219	−3.27
Sagittatoside A or icariin	C33H40O15	[M + H]+	677.2440	13.85	677.2419	−3.10	13.84	677.2421	−2.81
**ESI (-) mode**									
Bergapten or isobergaptene	C12H8O4	[M − H]-	215.0350	0.81	215.0336	−6.51	0.85	215.0333	−7.91
Epimedoside A or ikarisoside B	C32H38O15	[M − H]-	661.2138	9.55	661.2157	2.87	9.55	661.2142	0.60
Icariside I or curcumin monoglucoside	C27H30O11	[M − H]-	529.1715	15.91	529.1729	2.65	15.9	529.1714	−0.19

HEP crude extract and treated serum in ESI (+) mode and in ESI (−) mode.

## Discussion

*Herba epimedii* (HEP) has long been used to treat bone disorders and its effects are possibly via modulating the circulating estradiol level (Xue et al., 2012). Flavonoids phytoestrogens have been proven to be the main active components of HEP and actions of HEP are mediated by ERs (Chen et al., 2011; Xiao et al., 2014). The present study confirmed the effectiveness of HEP extract on protecting bone against ovarian sex hormone deficiency and the osteoprotective activities of HEP were mediated by increasing circulating estradiol and inhibiting bone turnover in ovariectomized rats. Furthermore, we reported that the inhibitory activity of HEP on FSH might also participate in the beneficial actions of HEP on bone. Two-way ANOVA analysis indicated the interactions between HEP and two clinically prescribed SERMs (tamoxifen and raloxifene) were beneficial to the preventing of bone loss in mature OVX rats. Surprisingly, HEP neither induced undesirable effect nor altered the uterotrophic effects of SERMs in OVX rats. Results of *in vitro* study showed the direct estrogenic activity of HEP and the interaction between HEP and SERMs in human osteosarcoma MG-63 cells. To the best of our knowledge, our study is the first to investigate the potential herb-drug interactions between bone protective herb HEP and clinically prescribed SERMs.

The dosage of HEP used in the present study was selected based on our previous study (Xie et al., 2005). Our study showed that HEP at 0.16 g/kg.day was effective in protecting OVX rats from bone loss. The increase in circulating estradiol by HEP treatment was thought to be the sole reason for its bone protective activity in OVX rats (Xue et al., 2012). In addition to the stimulatory effect on circulating estradiol, we further reported the suppression effect of HEP on FSH, which might also be involved in the osteoprotective activity of HEP in OVX rats. In fact, increase in serum FSH level was found to begin even before the decrease in estrogen and such an increase in FSH is accompanied by a boost in bone markers in postmenopausal women (Sun et al., 2006), which is in consistence with our observation in OVX rats that the higher the circulating FSH is, the higher the bone turnover rate would become. However, the possible role of circulating LH in the development and treatment of postmenopausal bone loss is not well studied. Results of our present study confirmed the bone protective effects of SERMs and further found that SERMs also stimulated the circulating estradiol and inhibited circulating FSH levels in OVX rats. Our study is the first to compare the bone protective activities between HEP and SERMs and found that HEP exerted similar but weaker bone protective effect than SERMs in OVX rats in terms of the increase in BMD. However, this seems to be in conflict with our observation that the circulating levels of estradiol and FSH in OVX rats treated with HEP were comparable to those in either the tamoxifen or raloxifene groups. These results suggest that HEP acts differently from SERMs and the regulation on hypothalamus-pituitary-gonadal axis (HPG) could not fully explain the antiosteoporotic effects of HEP.

A recent study has indicated that it is the local estradiol, rather than the circulating level of estradiol, that determines the estrogenic effects of estradiol and estradiol-like compounds in tissues (Huhtinen et al., 2012). HEP is a mixture of phytoestrogens and these phytoestrogens mediate the estrogenic activities of HEP via estrogen receptors (Xiao et al., 2014). Phytoestrogens have been reported to facilitate the clearance of estradiol from local tissues and enhance the catalysis of estradiol to more benign metabolites (Wood et al., 2007). Moreover, phytoestrogens, which exert both ER agonistic and antagonistic activities depending on the tissue types (Lecomte et al., 2017), have been demonstrated to be dependent on their own concentrations as well as the estradiol concentrations in local environments (Kim and Park, 2012). Based on these findings, HEP probably regulates the clearance of estrogens from reproductive tissues and (or) the catalysis of estradiol, which in turn determines the agonistic or antagonistic activities of phytoestrogens in local tissues and leads to diverse activities. Uterus is one of the estrogen sensitive tissues and also the tissue where the side effects of HRT and SERMs are often reported (Morello et al., 2002). Our results showed that HEP did not alter the weight of uterus in OVX rats despite its stimulatory effect on circulating estradiol; this provides further supporting evidence for our prediction that HEP acts differently from SERMs possibly due to the regulation of estradiol metabolism by the phytoestrogens it contains. Further investigation regarding the actions of HEP on local estradiol metabolism is needed.

HEP exerts estrogenic activities via ERs and activates the phosphorylation of ERα in an ERE-dependent manner (Xiao et al., 2014). As activities of SERMs are also mediated by ERs, it is necessary to investigate whether HEP interacts with SERMs to decrease or increase their activities, a point that is of special importance for those postmenopausal women who are taking HEP in addition to their standard treatment with SERMs. Results of two-way ANOVA analysis indicated the existence of interactions between HEP and SERMs on bone and uterus, while cotreatment with HEP did not alter the efficacy of either tamoxifen or raloxifene in bone or worsen their side effects in the uterus of OVX rats. *In vitro* study confirmed the presence of interactions between HEP and SERMs by using metabolically activated HEP. Cotreatment of SERMs with metabolically activated HEP reduced the stimulatory activity of tamoxifen at 10^−12^ M on ALP activities in MG-63 cells but did not alter the activities of raloxifene at all concentrations and tamoxifen at most of the concentrations applied. Based on our experience that the levels of the major chemical markers and metabolites of TCMs were too low to be detected in the serum of TCM-treated animals, HEP at a high dosage, which was 10 times of its dosage that was used for *in vivo* study, was used for harvesting metabolically activated HEP for the *in vitro* studies in the present study. We observed that metabolically activated HEP could blunt the stimulatory activity of tamoxifen at 10^-12^ M in MG-63 cell, but not in OVX rats, which was likely to be due to the high dosage of HEP used in preparing the HEP-treated serum. Therefore, the *in vivo* study should be of more relevance since HEP interacted with SERMs on bone without altering their activities.

It is of interest to note that the crude HEP extract, but not the metabolically activated HEP, stimulated ERE activities in MG-63 cells, suggesting that the action of biologically activated HEP might be different from that of the crude HEP extract. It is possible that some functional chemicals that were originally in the HEP extract might have been metabolized in the HEP-treated serum, which in turn produced different active chemical profiles between the HEP extract and the metabolized HEP. In fact, icariin, a prenylated flavonoid, has been demonstrated to be the main bioactive phytochemical that predominantly mediates the actions of HEP (Li et al., 2015). A previous study from our team has demonstrated that icariin could activate the phosphorylation of ER but it does not bind to ER or active ERE in rat osteoblastic UMR106 cells (Ho et al., 2018) or in human osteosarcoma MG-63 cells (Zhou et al., 2019). In agreement with our present results, icariin could only partially restore OVX-induced bone loss in OVX rats in comparison to estradiol (Zhou et al., 2019). Importantly, in the present study, icariin could be found in both the crude HEP and the metabolically activated HEP. Thus, it is believed that the activation of ERE-independent ER pathway by metabolically activated HEP *in vivo* might be attributable to the presence of icariin while the activation of ERE activity by the crude HEP might be a combined result of icariin and other unmetabolized chemicals in the crude HEP.

4-Hydroxy tamoxifen and endoxifen are the two most active forms of tamoxifen and their binding affinities to ERs are comparable to estrogen (Borgna and Rochefort, 1981). Moreover, tamoxifen shows equivalent affinity to both subtypes of ER (Kuiper et al., 1997). Compared with tamoxifen, phytoestrogens that are contained in HEP, like icariin and icaritin, show much weaker affinity to ERs (Tao et al., 2017; Ho et al., 2018), which makes them less competitive than tamoxifen in binding to ERs. Therefore, HEP and tamoxifen may show preference for different ER pathways, which might, at least partially, explain why HEP interacts with tamoxifen without affecting their activities on the bone and uterus of OVX rats.

**FIGURE 6:**
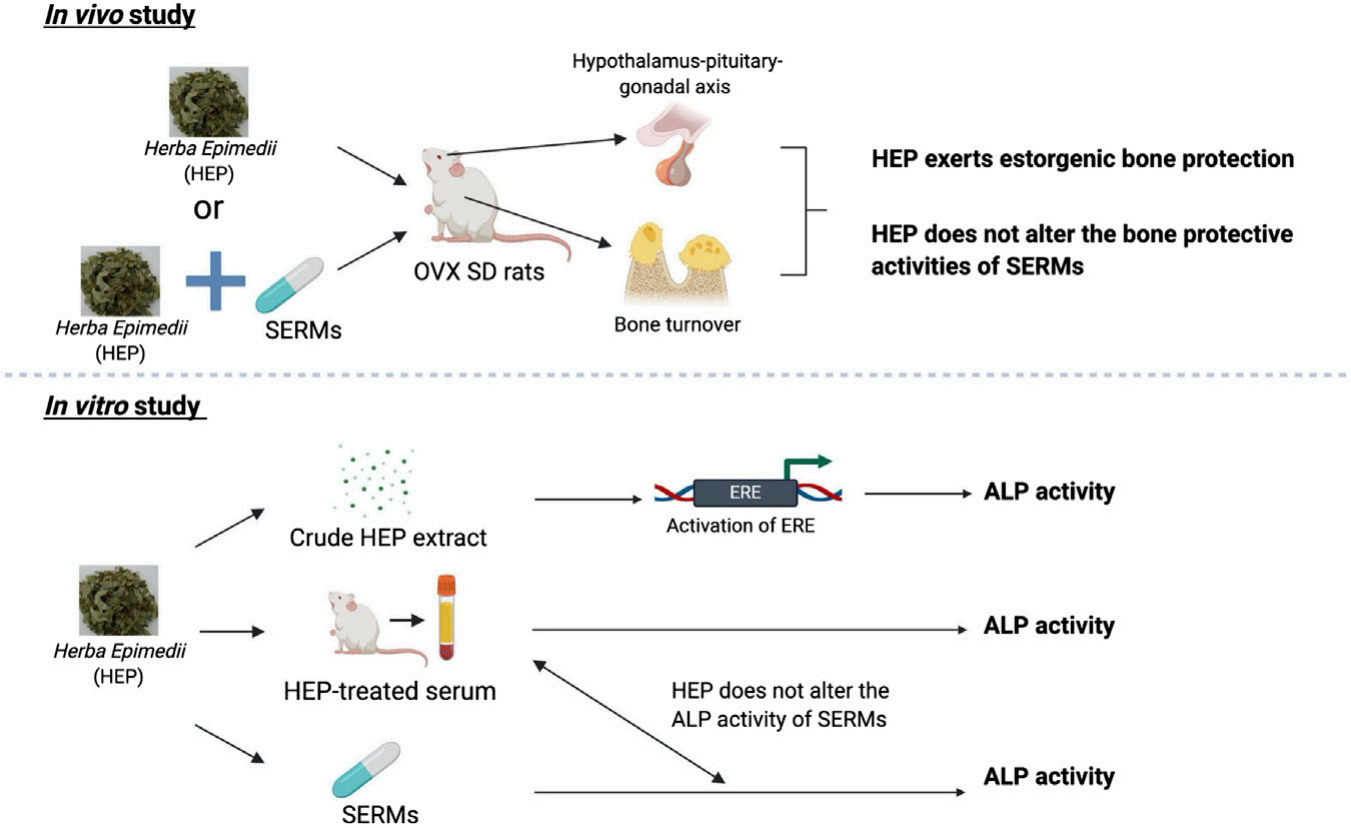
Bone protective activities of HEP alone and in combinations with SERMs. 1) HEP extract effectively protects against ovarian sex hormone deficiency-induced bone loss, possibly via its regulation of the hypothalamus-pituitary-gonadal axis without causing any undesirable effects on the uterus; 2) HEP interacts with SERMs on the bone and uterus without altering their responses to SERMs; 3) crude HEP extract, but not the metabolically activated HEP in the form of HEP-treated serum, directly increases ERE activities in human osteoblastic MG-63 cells, indicating the actions of the crude HEP might be different from those of the metabolically activated HEP.

As summarized in Figure 6, HEP extract effectively protects against ovarian sex hormone deficiency-induced bone loss, possibly via its regulation of the hypothalamus-pituitary-gonadal axis without causing any undesirable effects on the uterus. HEP interacts with SERMs on the bone and uterus without altering their responses to SERMs. Crude HEP extract, but not the metabolically activated HEP in the form of HEP-treated serum, directly increases ERE activities in human osteoblastic MG-63 cells, indicating the actions of the crude HEP might be different from those of the metabolically activated HEP. Indeed, this suggests that metabolically activated HEP that mimics the clinical administration of TCM might exert bone protection in an ERE-independent manner. These results provide insights for understanding the systemic actions of HEP and indicate that using HEP alone and in combination with SERMs appears to be effective and safe for treatment of menopausal symptoms, including postmenopausal osteoporosis. Future clinical investigations in terms of effectiveness, safety, and difference between individuals are needed to confirm the preclinical observations.

## Data Availability Statement

The raw data supporting the conclusions of this article will be made available by the authors, without undue reservation, to any qualified researcher.

## Ethics Statement

The animal study was reviewed and approved by the Hong Kong Polytechnic University Animal Subjects Ethics Subcommittee (ASESC Case: 15-16/31-ABCT-HMRF).

## Author Contributions

LZ and K-YW planned and performed the experiments and statistical analysis and wrote the manuscript. WY, CP, and HX helped with the performing and sample collection of animal experiment. C-OC and DM helped with the chemical analysis. YZ and M-SW conceived and supervised the experiments and finalized and revised the manuscript. All authors reviewed the manuscript.

## Funding

This work was supported by the Health and Medical Research Fund (HMRF) grant (Ref. no. 13143771) of Hong Kong, Essential Drug Research and Development (2019ZX09201004-003–032) from Ministry of Science and Technology of China, Hundred Talents Program from Shanghai Municipal Commission of Health and Family Planning (2018BR03), and Program of Shanghai Academic Research Leader (19XD1423800).

## Conflict of Interest

The authors declare that the research was conducted in the absence of any commercial or financial relationships that could be construed as a potential conflict of interest.

## Abbreviation

BMD, bone mineral density; BV/TV, bone volume/total volume; Conn.D, connective density; DPD, deoxypyridinoline; ER, estrogen receptors; ERE, estrogen response element; FSH, follicle stimulating hormone; HEP, *Herba* *Epimedii*; HPG, hypothalamus-pituitary-gonadal; HRT, hormone replacement therapy; LH, luteinizing hormone; OCN, osteocalcon; OVX, ovariectomy; SERMs, Selective Estrogen Receptor Modulators; SMI, structure model index; Tb.N, trabecular bone number; Tb.Th, trabecular bone thickness; Tb.Sp, trabecular bone separation; TCMs, traditional Chinese medicine; VOI: volume of interest
